# Global burden, geographic and sex disparities, and projections to 2050 for salivary gland cancer across G20 countries: a GLOBOCAN 2024 analysis

**DOI:** 10.3389/fonc.2026.1995714

**Published:** 2026-10-26

**Authors:** Wen Yang, Yuzhen Ma, Chunxia Shu

**Affiliations:** Department of Oral and Maxillofacial Surgery, Suining First People’s Hospital, Suining, Sichuan, China

**Keywords:** G20, global burden, GLOBOCAN database, human development index, mortality-to-incidence ratio, salivary gland cancer

## Abstract

**Background:**

Salivary gland cancer is uncommon, and neither its distribution nor its trajectory across the G20 has been well described. Using the GLOBOCAN 2024 case counts, we described the 2024 burden in the 19 G20 member economies and in four HDI aggregates, and examined HDI-related gradients in population-level proxies for survival.

**Methods:**

We analyzed the GLOBOCAN 2024 case counts for salivary-gland cancer (ICD-10 C07–C08) in the 19 G20 member economies and four HDI groups. Age-standardized incidence (ASIR) and mortality (ASMR) rates, 5-year prevalence, and the mortality-to-incidence (MIR) and prevalence-to-mortality (PMR) ratios were tabulated by country, HDI group and sex, and related to HDI using Spearman rank correlation. Projections to 2050 follow the GLOBOCAN 2024 Cancer Tomorrow default demographic scenario, applying the 2024 case counts and age-specific rates to United Nations population projections.

**Results:**

In 2024 the G20 recorded 37,812 cases and 12,661 deaths, 46.2% of them in China and India. At country level, the HDI–MIR correlation was not significant (r = −0.04, 95% CI −0.48 to 0.42; P = 0.88), although MIR was highest in the low-HDI group (0.53) and lowest in the very-high-HDI group (0.34) and PMR varied 3.9-fold across aggregates. Male MIR exceeded female MIR in 16 of the 17 economies for which the ratio was computable, the sole exception being Türkiye. Holding 2024 age-specific rates constant, deaths at ages ≥75 years would rise by 152.8% by 2050; growth would be largest in the low- and medium-HDI aggregates (+134.4% and +96.1%) and smallest in Japan (+8.4%).

**Conclusion:**

The G20 burden was highly heterogeneous. Across the global HDI aggregates the burden followed development level, with the highest MIR in the low-HDI group, but this ordering did not carry over to a country-level HDI–MIR correlation among the 19 member economies. Projected growth was concentrated in low- and medium-HDI settings, which argues for strengthening diagnostic capacity and survivorship measurement there.

## 1 Introduction

Salivary gland cancers are uncommon epithelial malignancies, and their rarity hides considerable biological heterogeneity. The category spans multiple histologic entities that differ in anatomic predilection, molecular features, recurrence behavior and prognosis (2–4), United States population-based data show wide incidence variation by subtype, sex, age, race and anatomic site (5, 6). Registries and global estimation systems aggregate these tumors under the ICD-10 C07–C08 top site, which makes international comparison possible but masks subtype-specific variation (7).

Most of what is known comes from institutional and national series. These are useful for management, but heterogeneous case mix and referral contexts limit cross-country comparison (8, 9). Population-based studies from the United Kingdom, Denmark, the Netherlands and Spain show that incidence and survival can be measured nationally, and they also expose differences in ascertainment, classification and observation periods (10–12). Treatment access and survival may in addition track social vulnerability and demographic characteristics. Whether the burden of salivary-gland cancer varies across countries along a national development gradient has mostly been examined in single national series rather than comparatively, and that omission matters: rare head-and-neck sites can remain underrepresented in global assessments, a gap also visible in broad global oncology reviews (13).

This study selects the G20 as its analytical framework. the 19 member economies together hold roughly two-thirds of the world population and a comparable share of global cancer incidence, so one policy-relevant comparison set covers both the largest case volumes and a wide range of health-system capacities (14). these economies span almost the full range of the Human Development Index, which lets a development gradient be examined within a single comparable reporting framework instead of across partially overlapping regional or income aggregates (15, 16). G20 health systems are comparatively well documented, so contrasts identified within this frame are more directly actionable for cancer-control policy than those drawn from world-region groupings. Existing comparative work on head-and-neck cancer has been framed by world region or income tier rather than by a specific policy forum, and salivary-gland cancer has not previously been examined across the G20 (10–12).

A comparative G20 assessment speaks to that gap. The 19 member economies differ widely in age structure, health-system capacity and Human Development Index (HDI), and together contribute a substantial share of the global cancer burden. Using the GLOBOCAN 2024 case counts from Cancer Today and Cancer Tomorrow, we set out to quantify 2024 incidence, mortality and 5-year prevalence across the 19 economies and four HDI aggregates; to describe sex- and age-specific differences in counts, age-standardized rates and the mortality-to-incidence ratio (MIR); to relate the age-standardized incidence rate (ASIR), age-standardized mortality rate (ASMR) and MIR to HDI, with the prevalence-to-mortality ratio (PMR) as a population-level survival proxy; and to project cases and deaths to 2050 under the default demographic scenario of fixed rates, together with annual-percentage-change (APC) trend scenarios in six high-incidence economies.

## 2 Materials and methods

### 2.1 Data source and study population

This was a descriptive, population-based, cross-national study, and it drew on GLOBOCAN 2024 estimates (Cancer Today and Cancer Tomorrow, version 1.0) published by the Global Cancer Observatory (GCO) of the International Agency for Research on Cancer (IARC) (14, 17).

Salivary gland cancer was defined by GLOBOCAN site code 2 (ICD-10 C07–C08; major and minor glands). The study population comprised the 19 G20 member economies with complete data (Argentina, Australia, Brazil, Canada, China, France, Germany, India, Indonesia, Italy, Japan, the Republic of Korea, Mexico, the Russian Federation, Saudi Arabia, South Africa, Türkiye, the United Kingdom and the United States) together with the four GLOBOCAN HDI aggregate groups (codes 981–984) (15). The G20 nominally has 21 members, but the European Union and the African Union are supranational groupings rather than individual economies, so both were excluded and country-level analysis was restricted to the 19 sovereign member economies.

Case and death counts are the only outputs GLOBOCAN 2024 supplies; it publishes no 95% uncertainty intervals (UIs) for them. Every table, figure and statistic in this paper is built on the single GLOBOCAN 2024 release and its case counts, and presents ASIR, ASMR and 5-year prevalence as point estimates (18); the projection figure (Section 2.4) is likewise drawn from the GLOBOCAN 2024 Cancer Tomorrow baseline.

### 2.2 Variables, derived indicators, and units of analysis

Incident cases, deaths, 5-year prevalent cases, ASIR, ASMR and the 5-year crude prevalence rate were extracted for each country, HDI group and sex. All rates are standardised to the 1966 Segi–Doll World Standard Population and expressed per 100,000 (18). Neither MIR nor PMR can serve as a direct index of survival, because both are readily distorted by registry under-ascertainment, by heterogeneity in the distribution of histological subtypes, by diagnostic delay and by the natural history of the disease.

Derived ratios. For each country-sex-HDI stratum we computed the mortality-to-incidence ratio (MIR = all-age deaths/incident cases) as a case-fatality proxy, the prevalence-to-incidence ratio (PIR = 5-year prevalent cases/incident cases) as a burden proxy, and the prevalence-to-mortality ratio (PMR = 5-year prevalent cases/deaths) as an exploratory survival-pool indicator. All three are all-age quantities and are therefore sensitive to differences in population age structure between countries. The age-standardized rates (ASIR, ASMR) are reported alongside them for that reason, and country rankings by MIR were checked against the corresponding ASMR ranking to confirm that no ranking rested on age composition alone.

The country (n = 19) served as the unit of analysis for cross-country correlation and the HDI aggregate (n = 4) for stratified burden comparison; sex- and age-specific patterns were tabulated across five age bands (0–19, 20–39, 40–64, 65–74 and ≥75 years).

### 2.3 Correlation analysis

Country-level associations between continuous HDI values and MIR were assessed with two-sided Spearman rank correlations across the 19 member economies. Every numerical value in the text was checked against the GCO export for the corresponding field, and the country-level rates and ratios are those tabulated in the corresponding tables; where the text and a table could differ by rounding, the table governs (**Tables 1**, **2**). Country HDI values were taken from the Global Cancer Observatory 2024 release, the same source as the burden estimates and therefore internally consistent with the four HDI aggregate groups; these values may differ slightly from the most recent UNDP Human Development Report (15), and all HDI-based groupings in this paper use the GCO values. A prespecified sensitivity analysis excluded the four economies with reconstructed both-sexes counts (Section 2.5). Associations of HDI with ASIR and ASMR were assessed the same way, with Pearson correlations and sex-stratified analyses as further sensitivity checks. We report the Spearman rank correlation coefficient (r), two-sided P values, sample sizes and changes in r between analyses. Because GLOBOCAN 2024 publishes no variance estimates for age-standardized rates and no uncertainty intervals for case and death counts, 95% confidence intervals for every reported correlation were obtained by non-parametric percentile bootstrap with 20,000 resamples (fixed seed), and the Fisher z-transformation is reported as a secondary check. A bias-corrected and accelerated (BCa) interval was not used: BCa also requires a jackknife estimate of the acceleration constant, which is not identified for a rank statistic computed on 19 observations, so the percentile method is the one actually applied. The median bias of each bootstrap distribution was inspected as a check on its centring. With n = 19 or fewer in every case, all intervals are wide, and the coefficients describe the direction of association rather than precise effect sizes. Many correlation tests were run and no formal correction for multiple comparisons was applied, so nominally significant P values should be read cautiously to avoid type-I error inflation.

**Table 1:** Salivary-gland cancer incidence, mortality, and 5-year prevalence in 2024 by country and HDI aggregate (both sexes, all ages).

Country/Group	Incidence		Mortality		Prevalence				
	Number of cases	ASIR (per 100,000)	Number of cases	ASMR (per 100,000)	Number of cases	Crude rate (per 100,000)	MIR	PIR	PMR
Argentina	429	0.67	127	0.17	1,409	3.1	0.30	3.28	11.09
Australia	387	0.80	127	0.17	1,411	5.3	0.33	3.65	11.11
Brazil	1,577	0.54	508	0.16	4,681	2.2	0.32	2.97	9.21
Canada	695	0.86	165	0.15	2,456	6.2	0.24	3.53	14.88
China	10,676	0.47	3,735	0.14	33,716	2.4	0.35	3.16	9.03
France (metropolitan)	890	0.65	305	0.14	3,056	4.6	0.34	3.43	10.02
Germany	1,107	0.53	444	0.16	3,910	4.6	0.40	3.53	8.81
India	6,791	0.45	2,033	0.14	17,842	1.2	0.30	2.63	8.78
Indonesia	2,355	0.75	1,278	0.40	6,706	2.4	0.54	2.85	5.25
Italy	1,126	0.84	433	0.20	3,879	6.5	0.38	3.44	8.96
Japan	1,913	0.69	738	0.17	6,719	5.4	0.39	3.51	9.10
Korea, Republic of	653	0.73	172	0.14	2,423	4.7	0.26	3.71	14.09
Mexico	671	0.46	193	0.12	2,084	1.6	0.29	3.11	10.80
Russian Federation	1,447	0.57	655	0.25	4,724	3.3	0.45	3.26	7.21
Saudi Arabia	87	0.30	35	0.13	315	0.9	0.40	3.62	9.00
South Africa	276	0.43	78	0.13	799	1.2	0.28	2.89	10.24
Türkiye	575	0.51	153	0.13	1,977	2.3	0.27	3.44	12.92
United Kingdom	737	0.58	311	0.16	2,685	3.9	0.42	3.64	8.63
United States of America	5,420	0.84	1,171	0.14	19,243	5.6	0.22	3.55	16.43
Very-high-HDI group	20,701	0.71	7,031	0.26	39,251	4.8	0.34	1.90	5.58
High-HDI group	19,611	0.52	7,475	0.17	60,188	2.2	0.38	3.07	8.05
Medium-HDI group	10,265	0.44	3,730	0.17	26,911	1.1	0.36	2.62	7.21
Low-HDI group	4,635	0.63	2,470	0.34	5,032	0.8	0.53	1.09	2.04

ASIR, age-standardized incidence rate; ASMR, age-standardized mortality rate; HDI, Human Development Index (aggregate group); MIR, mortality-to-incidence ratio (all-age deaths/all-age incident cases); PIR, prevalence-to-incidence ratio (5-year prevalent cases/incident cases); PMR, prevalence-to-mortality ratio (5-year prevalent cases/deaths). ASIR and ASMR are per 100,000 person-years, standardized to the 1966 Segi–Doll World Standard Population and taken from the GLOBOCAN 2024 export for the corresponding field; for the five economies for which the export publishes no both-sexes all-ages record (France, Germany, the Republic of Korea, South Africa and the United States), the both-sexes rate was reconstructed by summing the male and female age-specific deaths and populations and re-standardizing against the same standard population; the 5-year prevalence rate is a crude all-ages rate. Data are point estimates only; GLOBOCAN provides no public 95% uncertainty intervals for age-standardized rates or derived ratios. Rates and ratios are shown to two decimal places. Rounding may cause displayed values to differ marginally from ratios recomputed from the rounded counts. For France, Germany, Republic of Korea and the United States (all Very-high-HDI), selected public Both-sexes 2024 counts were unavailable in the GLOBOCAN 2024 export and were reconstructed as the sum of the complementary Male and Female sub-tabulations (deaths only: France and the Republic of Korea; 5-year prevalent cases only: the United States; both: Germany); derived ratios were then recomputed from the merged counts (see Section 2.5 and **Supplementary Table S2**). Source: GLOBOCAN 2024.

**Table 2:** Distribution of salivary-gland cancer deaths, ASMR, and MIR in 2024, and projected deaths by 2050, across age groups, G20 regions, and HDI aggregates.

Characteristic, y	Females						Males						Both sexes					
	ASMRa (2024)	MIR^b^	No. of deaths in 2024	Change, %	No. of deaths in 2050	Change, %	ASMRa (2024)	MIR^b^	No. of deaths in 2024	Change, %	No. of deaths in 2050	Change, %	ASMRa (2024)	MIR^b^	No. of deaths in 2024	Change, %	No. of deaths in 2050	Change, %
0-19	0.00	0.15	67	-1.49	66	-1.5	0.01	0.22	111	-2.70	108	-2.7	0.00	0.18	178	-2.25	174	-2.2
20-39	0.06	0.17	547	10.60	605	10.6	0.04	0.20	544	12.32	611	12.3	0.05	0.18	1,091	11.37	1,215	11.4
40-64	0.19	0.29	2,642	30.32	3,443	30.3	0.45	0.36	5,222	35.13	7,055	35.1	0.31	0.33	7,864	33.54	10,498	33.5
65-74	0.63	0.38	1,714	56.48	2,682	56.5	1.41	0.47	3,361	62.59	5,464	62.6	1.01	0.42	5,075	60.45	8,146	60.5
≥75	1.44	0.55	2,618	143.24	6,367	143.2	2.92	0.52	3,881	159.24	10,061	159.2	2.02	0.55	6,499	152.79	16,429	152.8
Region																		
Argentina	0.10	0.27	47	59.57	75	59.6	0.15	0.31	80	82.50	146	82.5	0.17	0.29	127	73.23	220	73.2
Australia	0.12	0.24	38	102.63	77	102.6	0.26	0.39	89	98.88	177	98.9	0.17	0.32	127	100.00	254	100.0
Brazil	0.13	0.27	191	112.57	406	112.6	0.23	0.33	317	100.32	635	100.3	0.16	0.30	508	105.12	1,042	105.1
Canada	0.11	0.21	52	75.00	91	75.0	0.23	0.32	113	87.61	212	87.6	0.15	0.27	165	83.64	303	83.6
China	0.09	0.31	1,282	81.59	2,328	81.6	0.16	0.36	2,453	63.62	4,013	63.6	0.14	0.34	3,735	69.79	6,341	69.8
France (metropolitan)	0.11	0.29	104	49.04	155	49.0	0.23	0.36	201	56.22	314	56.2	0.14	0.33	305	53.44	468	53.4
Germany	0.14	0.38	160	27.50	204	27.5	0.25	0.38	284	37.68	391	37.7	0.16	0.38	444	34.01	595	34.0
India	0.09	0.24	674	88.72	1,272	88.7	0.16	0.32	1,359	93.23	2,626	93.2	0.14	0.28	2,033	91.73	3,898	91.7
Indonesia	0.22	0.48	424	66.75	707	66.7	0.59	0.60	854	81.73	1,552	81.7	0.40	0.54	1,278	76.84	2,259	76.8
Italy	0.13	0.27	149	36.24	203	36.2	0.29	0.40	284	48.24	421	48.2	0.20	0.34	433	44.11	624	44.1
Japan	0.13	0.37	266	10.15	293	10.2	0.23	0.39	472	7.63	508	7.6	0.17	0.38	738	8.40	800	8.4
Korea, Republic of	0.12	0.23	60	80.00	108	80.0	0.21	0.32	112	75.89	197	75.9	0.14	0.28	172	77.33	305	77.3
Mexico	0.10	0.23	76	131.58	176	131.6	0.17	0.29	117	115.38	252	115.4	0.12	0.26	193	121.76	428	121.8
Russian Federation	0.13	0.29	250	31.20	328	31.2	0.37	0.56	405	9.63	444	9.6	0.25	0.45	655	17.86	772	17.9
Saudi Arabia	0.04	0.20	5	60.00	8	60.0	0.16	0.47	30	106.67	62	106.7	0.13	0.40	35	100.00	70	100.0
South Africa	0.11	0.21	29	89.66	55	89.7	0.16	0.32	49	91.84	94	91.8	0.13	0.26	78	91.03	149	91.0
Türkiye	0.11	0.29	72	81.94	131	81.9	0.15	0.26	81	114.81	174	114.8	0.13	0.27	153	99.35	305	99.3
United Kingdom	0.11	0.27	90	44.44	130	44.4	0.22	0.45	221	56.11	345	56.1	0.16	0.36	311	52.70	475	52.7
United States of America	0.09	0.22	411	57.42	647	57.4	0.17	0.24	760	76.58	1,342	76.6	0.14	0.23	1,171	69.80	1,988	69.8
HDI																		
Very high	0.13	0.28	2,505	46.83	3,677	46.8	0.26	0.38	4,526	53.50	6,946	53.5	0.26	0.34	7,031	51.12	10,623	51.1
High	0.13	0.29	2,637	76.57	4,658	76.6	0.25	0.38	4,838	71.36	8,292	71.4	0.17	0.38	7,475	73.15	12,950	73.2
Medium	0.12	0.27	1,424	94.03	2,763	94.0	0.21	0.38	2,306	97.40	4,552	97.4	0.17	0.36	3,730	96.11	7,314	96.1
Low	0.29	0.46	1,021	131.00	2,358	131.0	0.43	0.58	1,449	136.87	3,432	136.9	0.34	0.53	2,470	134.41	5,790	134.4

a: ASMR: age-standardized mortality rate (per 100,000 person-years, World Standard Population); b: MIR: mortality-to-incidence ratio (all-age deaths/all-age incident cases); HDI: Human Development Index aggregate group; G20: Group of Twenty. Region rows are the 19 G20 member countries; HDI rows are the four global HDI aggregate groups; age rows are world totals by age band. Projected deaths refer to 2050 under the GLOBOCAN Cancer Tomorrow default demographic scenario (2024 baseline), in which future burden changes only through population growth and aging; the % change is identical for 2024 and 2050 columns because the projection applies 2024 age-specific rates to the projected population. Each sex block carries a single ASMR column, labelled ASMRa (2024), together with a separate MIR column, so the age-group rows give one ASMR value and one MIR value per sex rather than two ASMR values. Because age-specific rates are held fixed at their 2024 values, ASMR is identical in 2024 and 2050 by construction and only the death counts change; the two adjacent values in the 65–74-year female row (0.63 and 0.38) is its ASMR and its MIR. Data are point estimates; GLOBOCAN provides no public 95% uncertainty intervals for these indicators. ASMRs and MIRs are shown to two decimal places and percentage changes to two decimal places; rounding may cause minor discrepancies with values computed from the counts. The both-sexes ASMR column is identical to the corresponding column of **Table 1** and is standardised in the same way: where the GCO export publishes a both-sexes all-ages rate it is used directly, and where it does not the rate was reconstructed from the male and female age-specific counts and person-populations re-standardised to the World Standard Population. Source: GLOBOCAN 2024.

### 2.4 Projection to 2050

Projected case and death count through 2050 were taken from the GLOBOCAN 2024 Cancer Tomorrow under the 2024 baseline static-rate demographic scenario (19). A single data vintage is used throughout this paper: every burden estimate, every HDI comparison, every correlation, the primary 2050 projection and the APC scenario analysis of this section all rest on the GLOBOCAN 2024 case counts, so no figure reported here mixes releases, and the 2024 count is the anchor for every extrapolation in the paper. This scenario applies the 2024 age-specific rates to United Nations World Population Prospects 2024 projections (20), it captures population growth and aging only. Percent changes between 2024 and 2050, calculated as [(N2050 − N2024)/N2024] × 100%, are reported descriptively for selected countries and HDI aggregates. These figures describe demographic pressure under a fixed-rate scenario; they are not a forecast of what will occur and not a clinical forecast for any patient or institution, and they assume no change in risk factors, screening, diagnostic practice or treatment.

Annual-percentage-change (APC) scenario analysis of incident cases was performed for the six G20 economies with the highest 2024 both-sexes ASIR, listed in the caption of the accompanying projection figure. APC scenario analysis is a transparent trend-extrapolation approach: a fixed annual rate of change is compounded onto the baseline count, N(t) = N2024 × (1 + APC)^(t−2024), with no covariate assumptions. The GLOBOCAN 2024 count serves as the anchor, so the baseline is the same 2024 case count used everywhere else in this paper and the scenario series are directly comparable with the primary projection. The APC scenarios are therefore offered as illustrative sensitivity envelopes rather than as an alternative primary projection, which remains the 2024-based demographic projection reported in the Abstract, Results and Conclusion. Scenarios from −3% to +3% in 1% steps, plus the 0% baseline, were run for 2024–2050. Country selection followed the 2024 ASIR ranking alone and was not strictly prespecified; registry coverage varies across these economies, and the Indonesian estimates in particular rest substantially on modelled values, so the corresponding scenario should be read with caution. Because the anchor and the projection alike rest on the GLOBOCAN 2024 counts, the two sets of figures are directly comparable, and every derived percentage reported below was recomputed against the tabulated values (**Tables 1**, **2**).

### 2.5 Back-filling of missing cells and sensitivity analysis

For 2024, selected Both-sexes counts for France, Germany, the Republic of Korea and the United States were unavailable in the GCO export and were reconstructed as the sum of the complementary Male and Female sub-tabulations: deaths for France and the Republic of Korea, 5-year prevalent cases for the United States, and both for Germany. Derived ratios were recalculated from the merged counts, and these cells are flagged as reconstructed; the HDI values used throughout and the underlying Male, Female and reconstructed Both-sexes values are listed in full (**Supplementary Tables S1**, **S2**). A separate and unrelated coverage limitation applies to the four global HDI aggregate rows: for the very-high-HDI and low-HDI groups the GCO export published mortality and 5-year prevalence as Male-only sub-tabulations, so the both-sexes aggregate values were obtained by adding the Male and Female rows. In the primary analysis (n = 19), HDI showed no association with MIR (r = −0.04, 95% CI −0.48 to +0.42; P = 0.881); both the coefficient and its P value are computed on the full-precision HDI values published with the GLOBOCAN 2024 release and reproduce to r = −0.04, P = 0.881 (**Supplementary Table S1** rounds the index to three decimals, and recomputing from those rounded values gives r = −0.02, P = 0.93). Excluding these four economies (n = 15) left the association non-significant and reversed its sign (r = +0.07, 95% CI −0.46 to +0.56; P = 0.800; Δr = 0.108); neither estimate differs from zero, and because Δr is far smaller than the width of either interval (about 0.9), the change of sign does not represent a substantive perturbation of the primary result. Every correlation reported in this paper is accompanied by a percentile-bootstrap 95% confidence interval (Section 2.3). The back-filling procedure assumes that all cases can be classified by sex and ignores cases with unknown sex, which may introduce minor bias in the derived MIR and PMR values; the same assumption applies to the very-high-HDI and low-HDI aggregate rows described above.

## 3 Results

### 3.1 Country-level variation in age-standardized incidence and mortality and sex-specific HDI associations

Age-standardized rates varied widely across the G20. ASIR ranged from 0.30 per 100,000 (Saudi Arabia) to 0.86 (Canada), with the three highest rates in Canada, Italy and the United States (0.86, 0.84 and 0.84) and the three lowest in Saudi Arabia, South Africa and India (0.30, 0.43 and 0.45) (**Figure 1A**; **Table 1**). ASMR ranged from 0.12 (Mexico) to 0.40 (Indonesia), the highest rates falling in Indonesia, the Russian Federation (0.25) and Italy (0.20), and the lowest in Mexico (0.12), Saudi Arabia, South Africa and Türkiye (0.13) (**Figure 1B**; **Table 1**). All ASIR, ASMR and prevalence values quoted in this section are read directly from the tabulated values and are given to the same two decimal places; where a value is derived from the tabulated counts rather than read from the table, the derivation is stated (**Table 1**).

**Figure 1:**
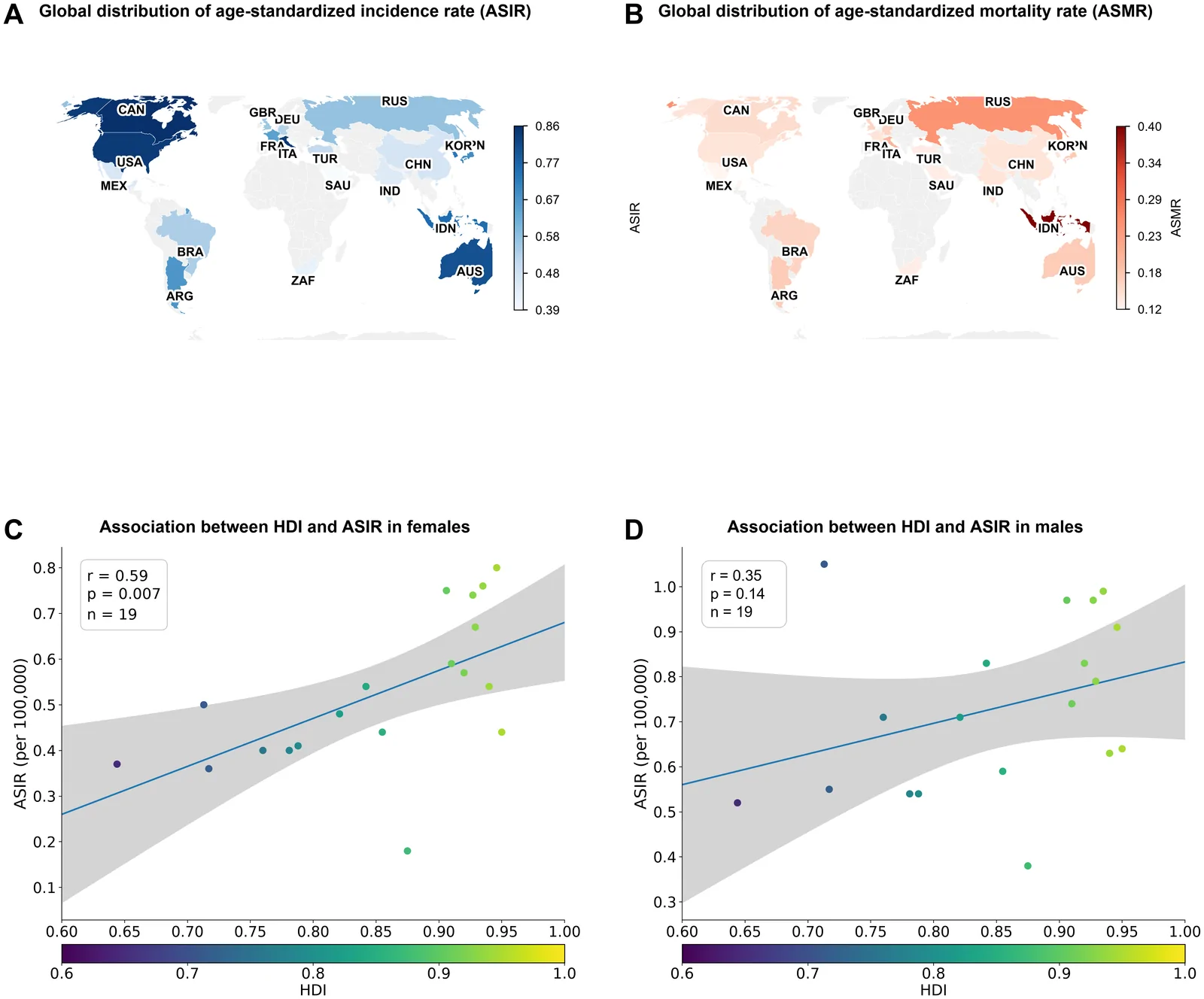
Age-standardized incidence and mortality rates of salivary-gland cancer by country in 2024 across the G20 economies, together with the sex-specific associations between HDI and ASIR. **(A)** Distribution of ASIR across the G20. **(B)** Distribution of ASMR across the G20. **(C)** HDI against female ASIR. **(D)** HDI against male ASIR. Source: GLOBOCAN 2024.

ASIR rose with HDI in the both-sexes analysis (r = 0.50, 95% CI 0.07 to 0.78, P = 0.028; n = 19), and the direction of association held in the sex-specific analyses (female r = 0.59, 95% CI 0.32 to 0.86, P = 0.007; male r = 0.35, 95% CI −0.17 to 0.76, P = 0.14), so that only the female association reaches significance. ASMR was not materially associated with HDI at the country level (r = 0.15, 95% CI −0.32 to 0.57, P = 0.53; n = 19). In the four global HDI aggregates, incidence was highest in the very-high-HDI group while mortality was highest in the low-HDI group; these four rows are global pools and not subsets of the G20, so this aggregate ordering is reported as a reference pattern rather than as a description of the member economies. The 5-year prevalence-to-mortality ratio (PMR) varied 3.9-fold across the HDI aggregates, from 2.04 in the low-HDI group to 8.05 in the high-HDI group, with the very-high-HDI group at 5.58. Across the 19 economies, HDI was strongly associated with the prevalence-to-incidence ratio (PIR: r = 0.91, 95% CI 0.77 to 0.96, P < 0.001) and weakly with PMR (r = 0.31, 95% CI −0.17 to 0.67, P = 0.20) (**Figures 1C, D**; **Table 1**).

### 3.2 Canada had the highest incidence and prevalence, whereas Indonesia had the highest mortality

Canada had the highest incidence ASR (0.86 per 100,000) and the highest 5-year prevalence ASR (3.19), followed for incidence by Italy and the United States (both 0.84) and Australia (0.80), and for prevalence by the United States (3.09), Italy (3.08) and Australia (3.05); the Republic of Korea (2.77) and Japan (2.57) took the next prevalence ranks on the prevalence axis, which reports the age-standardized prevalence rate rather than the crude prevalence rate (**Figure 2B**; **Table 1**). Indonesia recorded the highest mortality ASR (0.40), then the Russian Federation (0.25) and Italy (0.20); Germany (0.16) entered the top ten ahead of Canada (0.15) (**Figures 2A–C**).

**Figure 2:**
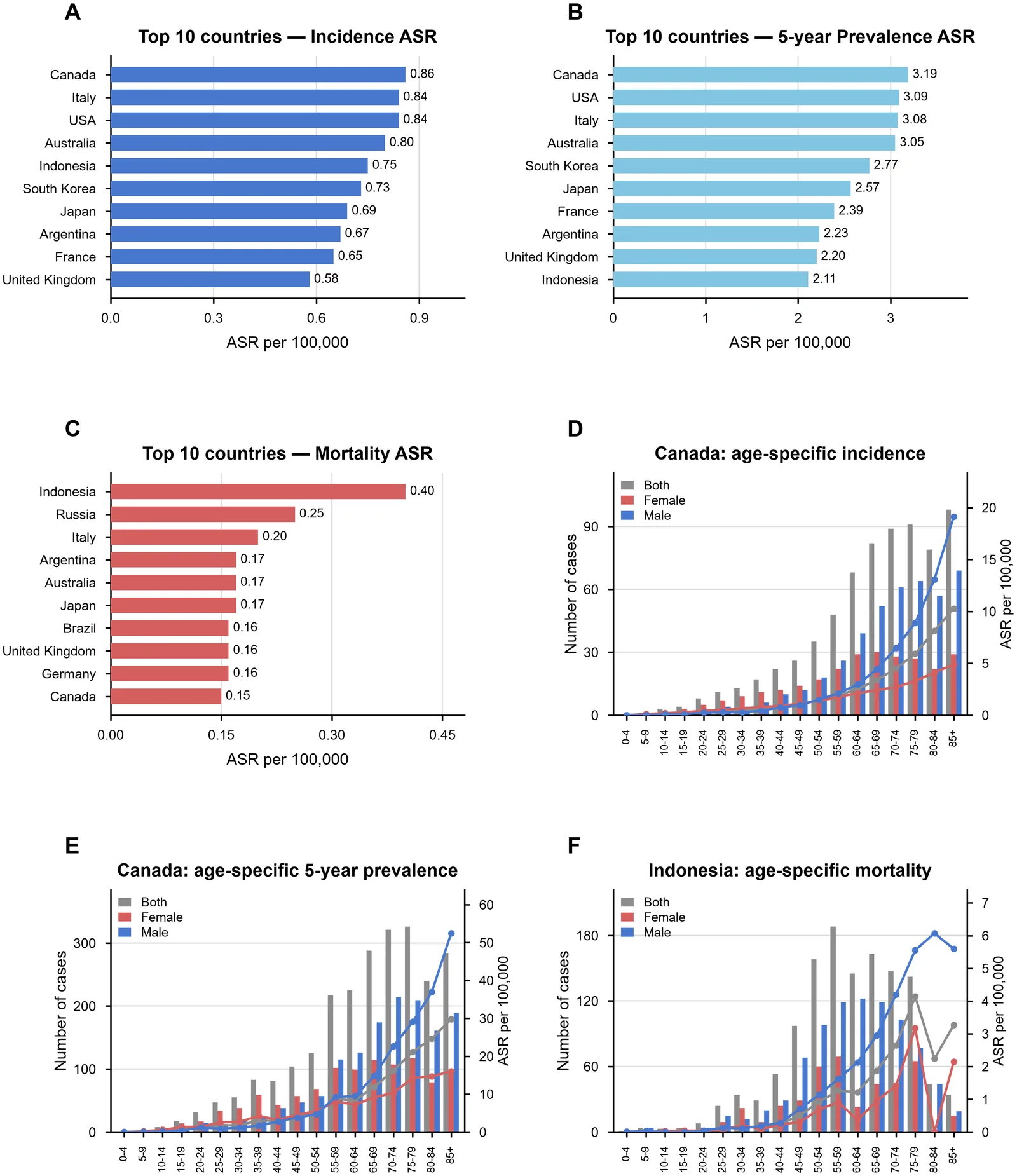
Top 10 G20 member-economies by age-standardized rates of incidence, 5-year prevalence and mortality for salivary-gland cancer in 2024, with age-specific patterns in Canada and Indonesia. Canada had the highest incidence and 5-year prevalence ASRs, whereas Indonesia had the highest mortality ASR. **(A)** Incidence ASR ranking. **(B)** 5-year prevalence ASR ranking. **(C)** Mortality ASR ranking. **(D)** Age-specific incidence in Canada. **(E)** Age-specific 5-year prevalence in Canada. **(F)** Age-specific mortality in Indonesia. Source: GLOBOCAN 2024.

Age-specific patterns differed by country and outcome. In Canada, the incidence curve is visibly right-skewed: it stays close to the axis until about age 40, then rises steeply and monotonically to a single peak in the 85-years-and-over band, roughly seventy times the level of the youngest band. The prevalence curve has a similar shape but peaks one band earlier, at 75–79 years, and is flatter on the right, the pattern expected of a shrinking pool of survivors at the oldest ages. In Indonesia, the mortality curve is bimodal rather than monotonic, with a first peak at 55–59 years and a second at 65–69 years; the male curve lies above the female curve across most of the age range, and the two converge only in the oldest bands (**Figures 2D–F**).

### 3.3 Sex- and age-specific disparities in incidence, mortality and prevalence

Male ASIR exceeded female ASIR in all 19 economies, the widest absolute gaps appearing in Indonesia (1.05 vs 0.50 per 100,000), Canada (0.99 vs 0.76) and the United States (0.97 vs 0.74); country-level 5-year prevalence rates follow the same ordering (**Figures 3A, B**). The same male excess held for ASMR in 18 of 19 countries (widest gaps: Indonesia 0.6 vs 0.2 and the Russian Federation 0.4 vs 0.1 per 100,000). MIR behaved a little differently: male MIR exceeded female MIR in 16 of the 17 economies for which the ratio was computable, the sole exception being Türkiye (male 0.2555 vs female 0.2791); the widest male excesses were in Saudi Arabia (0.48 vs 0.21) and the United Kingdom (0.54 vs 0.28) (**Figure 3C**; **Table 2**). Male and female MIR could not be computed for Australia and Brazil, because the export does not publish both the incidence and the mortality cell required for each sex. A complete country-by-sex MIR table is provided as **Supplementary Table S3**. In absolute deaths the male disadvantage was greatest in China (2,453 vs 1,282), India (1,359 vs 674) and Indonesia (854 vs 424).

**Figure 3:**
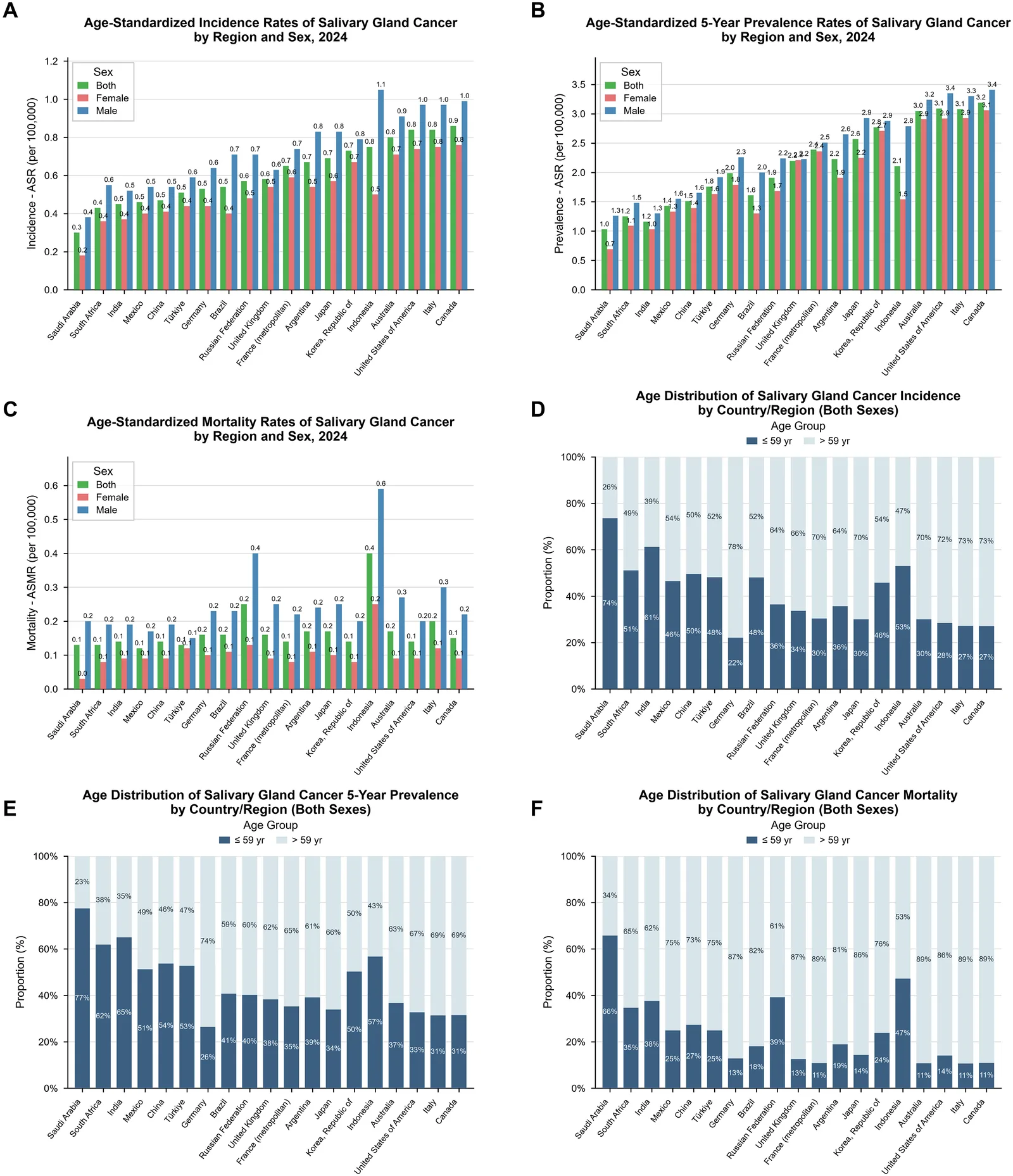
Age-standardized incidence rate (ASIR), 5-year prevalence rate, and age-standardized mortality rate (ASMR) of salivary-gland cancer across the 19 G20 economies in 2024, by sex **(A–C)**, and the age distribution of incidence, 5-year prevalence and mortality dichotomized at 59 years **(D–F)**. Countries are ordered by both-sexes ASIR. **(A)** Country ASIR by sex. **(B)** Country 5-year prevalence rate by sex. **(C)** Country ASMR by sex. **(D)** Age distribution of incident cases. **(E)** Age distribution of prevalent cases. **(F)** Age distribution of deaths. Source: GLOBOCAN 2024.

ASIR rose roughly 70-fold from the 0–19-year group to the ≥75-year group (0.05 to 3.6 per 100,000); ASMR rose from 0.01 to 2.0 and the age-specific 5-year prevalence rate from 0.1 to 10.5. Of the 20,707 world all-ages deaths in 2024 (a global total, not the G20 subtotal of 12,661), the 40–64, 65–74 and ≥75-year groups accounted for 38.0% (7,864), 24.5% (5,075) and 31.4% (6,499) respectively, and the two youngest groups for 6.1% (1,269); MIR increased from 0.1 to 0.6 (≥75). Persons aged 59 years and above represented 78% of cases in Germany, 73% in Canada and Italy, 70% in France, Japan and Australia and 64% in Argentina, against 49% in South Africa, 39% in India and 26% in Saudi Arabia. The 59-year cut is defined for these panels only and is not an age band of any tabulated grouping. Age-specific 5-year prevalence told a similar story: in Saudi Arabia (78%) and Indonesia (57%) more than half of prevalent cases were aged 59 years or younger, whereas in Germany (74%), Canada and Italy (69% each), the United States (67%), Japan (66%), France (65%) and Australia (63%) most prevalent cases were older. Mortality proportions concentrated still more heavily at older ages, exceeding 85% beyond age 59 in Italy, Australia, France and Canada (89% each), the United Kingdom and Germany (87% each), Japan and the United States (86% each), against 34% in Saudi Arabia, 53% in Indonesia and 62% in India (**Figures 3D–F**).

### 3.4 Correlations among mortality, incidence, MIR and projected temporal change

A higher MIR went with a higher ASMR across the G20 (r = 0.54, 95% CI 0.12 to 0.80, P = 0.016; n = 19), and absolute deaths rose almost linearly with incident cases (r = 0.99, 95% CI 0.97 to 1.00, P < 0.001; n = 19). The projected percentage increase in deaths by 2050 was inversely associated with baseline ASMR (r = -0.51, 95% CI -0.80 to -0.02, P = 0.044; n = 16); the corresponding inverse association with MIR (r = -0.54, 95% CI -0.81 to -0.05, P = 0.032; n = 16) was of similar magnitude (**Figures 4A–D**).

**Figure 4:**
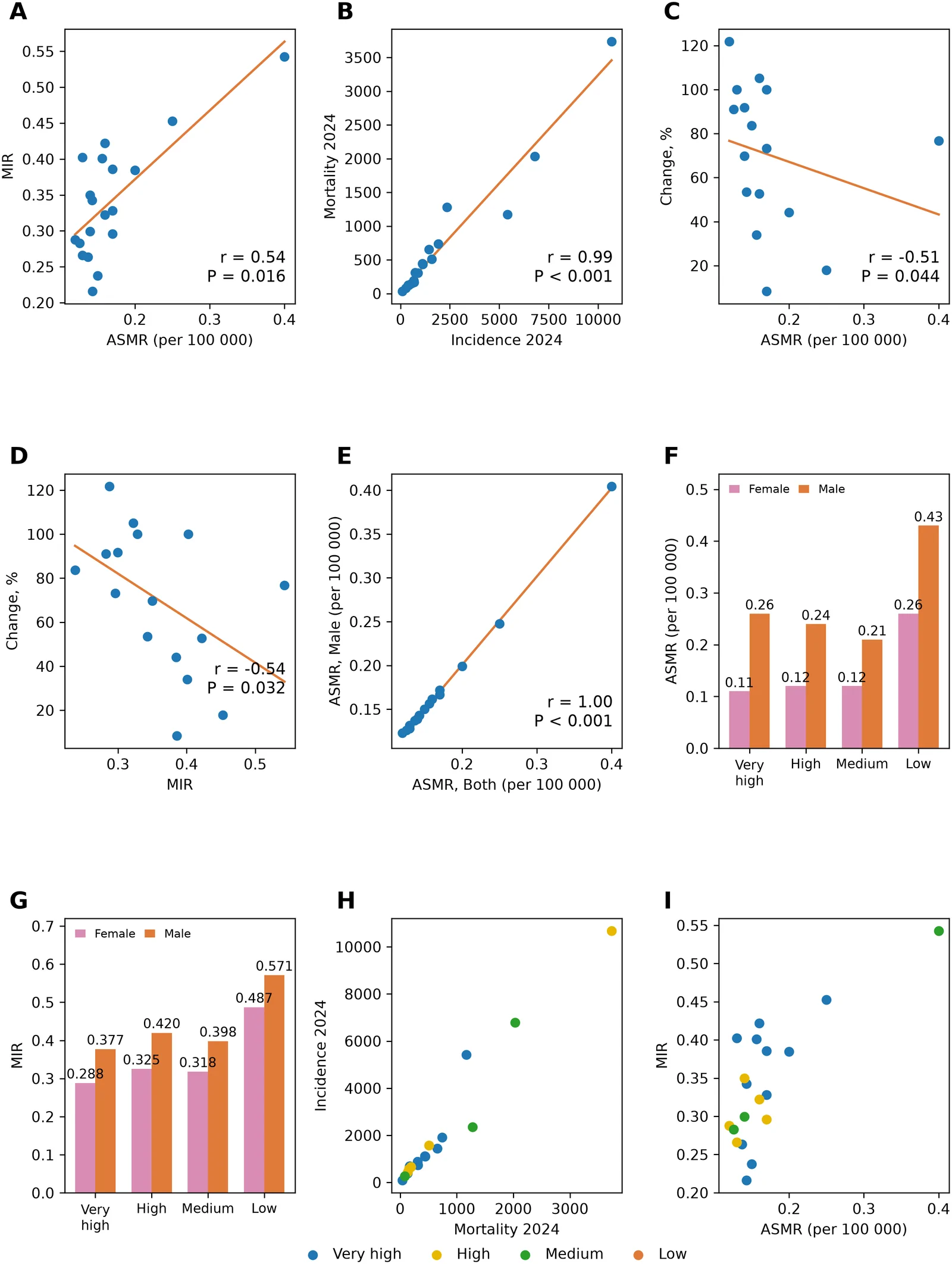
Correlations among MIR, age-standardized rates, burden counts and projected temporal change in salivary-gland cancer in 2024. **(A)** MIR versus ASMR. **(B)** Mortality counts versus incidence counts. **(C)** Percentage change versus ASMR. **(D)** Percentage change versus MIR. **(E)** Male ASMR versus both-sex ASMR. **(F)** Female and male ASMR by HDI group. **(G)** Female and male MIR by HDI group. **(H)** Incidence counts versus mortality counts, stratified by HDI group. **(I)** MIR versus ASMR, stratified by HDI group. Panels **(A, B)** re-present rate information already visualized in **Figures 2** and **3** in correlation-based plots; these panels are kept to display fitted association estimates alongside their corresponding confidence intervals. Panels **(E–G)** extend, rather than duplicate, the content of **Figure 3** by stratifying rates by sex and HDI group. For Panels **(H, I)**, countries were categorized into HDI groups according to UNDP thresholds using HDI values obtained from the Global Cancer Observatory database. Source: GLOBOCAN 2024. All correlation coefficients presented in Panels **(A–E)** are Spearman rank-order correlations. Panels **(A, B, E)** include the full set of 19 economies (n = 19), while Panels **(C, D)** are restricted to 16 economies with available projected percentage-change data up to 2050 (n = 16). Exact coefficients and sample sizes are reported in the main manuscript (Section 3.4). Panels **(F–I)** show grouped and stratified graphical outputs and are not described by a single correlation coefficient.

Male and both-sex ASMR were almost perfectly rank-correlated (r = 1.00, 95% CI 0.98 to 1.00, P < 0.001), though their absolute values differ because the both-sexes rate also includes females; male ASMR nonetheless stayed higher in every HDI stratum, rising from 0.26 to 0.43 per 100,000 in males and from 0.11 to 0.26 in females between the very-high- and low-HDI groups (**Figures 4E, F**).

The corresponding MIR was 0.38 in males and 0.28 in females in the very-high-HDI group, rising to 0.58 and 0.46 in the low-HDI group. Note that the HDI-aggregate ordering was not strictly monotone: male and female MIR were both slightly higher in the high-HDI group (0.38 and 0.29) than in the medium-HDI group (0.38 and 0.27), and the prevalence-to-mortality ratio peaked in the high-HDI group rather than in the low-HDI group. The substantive finding is therefore a difference across HDI aggregates rather than a monotone gradient. Crucially, this aggregate-level pattern did not reproduce at the country level: the HDI–MIR correlation across the 19 member economies was null (r = −0.04, 95% CI −0.48 to +0.42, P = 0.88; n = 19), and remained null, with the sign reversed, after the four economies with reconstructed both-sexes counts were excluded (n = 15, r = +0.07, 95% CI −0.46 to +0.56, P = 0.80) (**Figures 4G–I**).

### 3.5 Projected incidence under annual-percentage-change scenarios in six G20 economies

APC scenario projections of annual incident cases from 2024 to 2050 are shown for the six highest-ASIR economies, all anchored on the GLOBOCAN 2024 case count. Canada started from 695 incident cases in 2024, and the 0% scenario gives 695 in 2050, with +3% and −3% extremes of 1,499 and 315; Italy from 1,126 (0%: 1,126; extremes 2,428 and 510); the United States from 5,420 (0%: 5,420; extremes 11,689 and 2,455); Australia from 387 (0%: 387; extremes 835 and 175); Indonesia from 2,355 (0%: 2,355; extremes 5,079 and 1,067); and the Republic of Korea from 653 (0%: 653; extremes 1,408 and 296) (**Figures 5A–F**).

**Figure 5:**
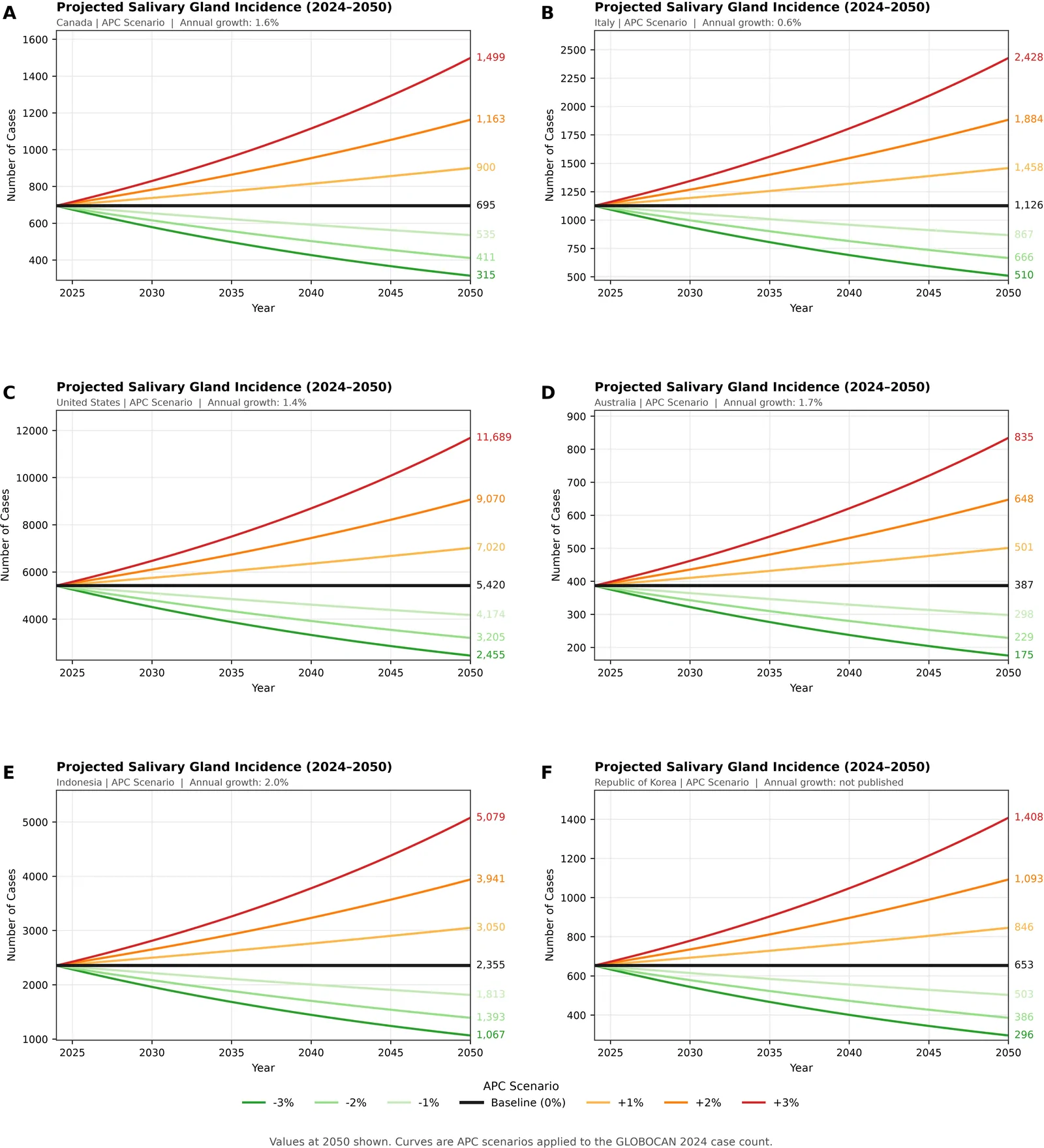
Projected annual incident cases of salivary-gland cancer from 2024 to 2050 under annual-percentage- in the six G20 economies with the highest 2024 both-sexes ASIR (Canada, Italy, the United States, Australia, Indonesia and the Republic of Korea), ordered by descending ASIR. Curves represent APC scenarios from −3% to +3% alongside the baseline (0%), with 2050 values shown. **(A)** Canada. **(B)** Italy. **(C)** United States. **(D)** Australia. **(E)** Indonesia. **(F)** South Korea. Source: GLOBOCAN 2024 Cancer Tomorrow baseline demographic projection (both sexes, incidence) with APC scenarios applied to the 2024 case count.

### 3.6 Sex-specific demographic projections of incidence and mortality through 2050

Projected both-sexes incidence rose steadily from 2025 to 2050, with no year-on-year decline in any of the six economies shown. China and India led with 13,568 and 11,413 cases by 2050, followed by the United States (7,735), Indonesia (3,968), Brazil (2,704) and Japan (1,823). Mortality followed the same ordering, at approximately 6.3 thousand deaths in China, 3.9 thousand in India and about 2.0 thousand in the United States by 2050 (**Figures 6A, B**).

**Figure 6:**
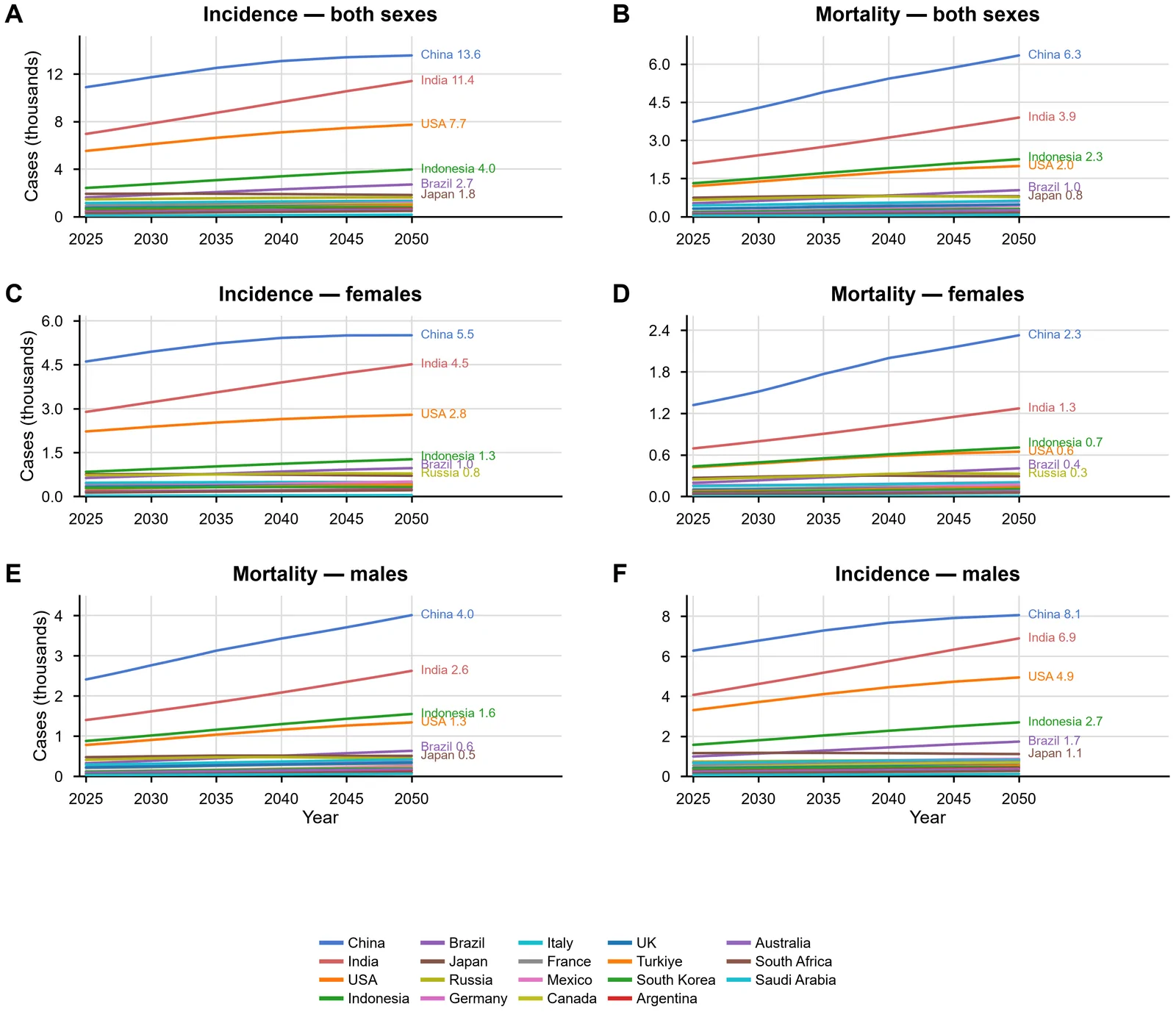
Projected annual numbers of incident and mortality cases of salivary-gland cancer from 2025 to 2050 by country and sex, based on the 2024 GLOBOCAN demographic projection. **(A)** Incidence in both sexes. **(B)** Mortality in both sexes. **(C)** Incidence in females. **(D)** Mortality in females. **(E)** Mortality in males. **(F)** Incidence in males. Source: GLOBOCAN Cancer Tomorrow (2024 baseline).

Among females, China and India again led with approximately 5.5 and 4.5 thousand incident cases by 2050 and corresponding female mortality of about 2.3 and 1.3 thousand. Male projections exceeded female projections, reaching approximately 4.0 and 2.6 thousand deaths and 8.1 and 6.9 thousand incident cases in China and India respectively (**Figures 6C–F**).

Between 2024 and 2050, projected mortality would rise most steeply where HDI is lowest: +134.4% in the low-HDI aggregate and +96.1% in the medium-HDI aggregate. Projected incidence increased as development level decreased, from +31.3% in the very-high-HDI group to +73.7% in the high-HDI group, +107.4% in the medium-HDI group and +124.9% in the low-HDI group. By age, the ≥75-year group would see the largest increase in world deaths (+152.8%, from 6,499 to 16,429). Country-level mortality increases were steepest for Mexico (+121.8%) and Brazil (+105.1%), with Australia and Saudi Arabia both at +100.0% and the smallest increase for Japan (+8.4%); Japan was the only economy projected to decline in incidence (−4.7%, from 1,913 cases in 2024 to 1,823 in 2050) (**Figures 6A–F**).

## 4 Discussion

Across the G20, 2024 MIR ranged from 0.22 (United States) to 0.54 (Indonesia) (18). China (10,676 cases) and India (6,791) account for 46.2% of G20 cases, yet the lowest MIRs occur in the very-high-HDI United States and Canada, and the highest age-standardized 5-year prevalence rates in Canada and Italy. Indonesia combines the highest ASMR (0.40) and MIR (0.54) with only fourth place in case count. Inter-country MIR disparities of this kind may partly reflect differences in healthcare access and diagnostic capacity, but they could equally arise from variable registry completeness and divergent histological subtype spectra. The comparison is ecological in two distinct senses: the unit of analysis is the country rather than the patient, a difference between national ratios does not license any inference about the survival probability of an individual patient in either country; and the ratio is computed from modelled national aggregates, it inherits whatever error the underlying registry and modelling steps carry. Statements about health-system performance below are therefore framed as hypotheses consistent with the observed ratios rather than as measurements of care quality. No causal conclusion follows from an ecological cross-sectional design, in which the unit of analysis is the population rather than the individual and group-level associations need not hold at the individual level (21, 22), the same limitation applies when a pattern is compared across two ecological units, and the ratio should be read as a descriptive national indicator rather than as a patient-level prognosis.

GLOBOCAN places salivary-gland cancer in the lower-incidence tier of head-and-neck malignancies, and in 2024 its ASIR was higher in higher-resource settings (18). Prognosis and treatment response vary substantially by subtype (3, 23), and top-site GLOBOCAN estimates pool all histologic types, the observed MIR range mixes detection quality with histology, historical US studies document comparable heterogeneity (5, 6). The difference in MIR across the four global HDI aggregate groups also did not translate into a statistically significant correlation at country level. That divergence between aggregate and country-level results is itself informative, because it warns against reading aggregate gradients as properties of member economies. The two levels are held strictly separate throughout: the aggregate rows are global pools covering all 186 GLOBOCAN populations, the country rows are the 19 G20 economies, and no statistic in this paper combines the two. Because the aggregates are population-weighted quantities on a different scale, an aggregate value outside the range of the country rows is expected rather than anomalous, and is not treated here as evidence about any individual economy.

Histology alone appears unlikely to explain the HDI gradient in MIR, although the present design cannot exclude it. Limited diagnostic capacity and later presentation would both be consistent with a higher MIR, and Indonesia’s profile (moderate ASIR, highest MIR) fits that reading. The weak positive HDI–ASIR correlation (r = 0.50, 95% CI 0.07 to 0.78, uncorrected P = 0.028) would be compatible with greater detection intensity in higher-HDI settings, though under-ascertainment in low-HDI registries could equally produce a correlation of this size, and the interval extends from a negligible to a strong association (14). Unequal access to specialized surgery, radiotherapy and multidisciplinary care probably contributes (24, 25): US cohorts link insurance status to stage and survival, with racial disparities (26); London data show ethnicity-specific incidence differences (27); and German and US cohorts identified socioeconomic disadvantage and comorbidity as adverse prognostic factors (28, 29). Taken at face value, MIR and PMR in this dataset appear to reflect unequal care pathways rather than markers of tumor biology. This is, however, an interpretation of the observed pattern rather than a direct measurement of care quality. The present data cannot disentangle true differences in case fatality from discrepancies in the completeness of case and death ascertainment. This inference is also indirect: the supporting evidence originates from within-country socioeconomic gradients and cannot be simply extrapolated to cross-country comparisons of the type examined here. Three non-mutually exclusive mechanisms may explain elevated MIR in lower-resource settings, and our data cannot discriminate between them. First, diagnostic delay and late presentation shift the stage distribution toward advanced disease and raise case fatality independent of underlying tumor biology. Second, restricted access to specialist treatment: salivary-gland cancer is primarily managed by surgical resection, often combined with adjuvant radiotherapy and systemic therapy for selected histology. In most low- and middle-income contexts, surgical and radiotherapy services are concentrated in a small number of specialized centers. Patients who cannot access these centers are counted as deaths in the MIR numerator without corresponding survival benefits. Third, under-ascertainment in cancer registries: if incident cases are systematically missed while deaths are captured via vital registration, or if both metrics are modelled from incomplete data, the calculated MIR will be inflated regardless of the true underlying case fatality. A fourth explanation cannot be ruled out: variation in the histologic mix of tumors across populations, which cannot be separated using top-level C07–C08 site estimates. These mechanisms are not mutually exclusive, and each implies distinct policy responses. Disentangling their relative contributions would require data on registry completeness and treatment access that are unavailable across all G20 economies.

Projected growth is concentrated in low- and medium-HDI G20 economies and is driven mainly by population aging rather than rising rates. The response these projections call for is structural rather than opportunistic: the capacity to diagnose and treat a slowly growing cohort of mostly older patients must be planned now, ahead of the demographic curve (13). Salivary gland tumors are rare and histologically diverse; their management therefore relies heavily on pathology, imaging, and specialized surgery that cannot be replaced by general oncology services. Rare tumors serve as a particularly informative stress test for health systems. Owing to small patient numbers, the fixed costs required to maintain pathology, imaging, and specialist surgical capacity cannot be distributed across a large patient volume. A health system that delivers good care for common cancers may still underperform for rare cancers, which thereby reveal underlying capacity limitations that would remain hidden when only high-volume malignancies are considered. Three structural intervention strategies follow directly and map onto the three mechanisms outlined above. (i) Expanding radiotherapy and surgical oncology capacity in lower-resource G20 economies, as resection completeness and delivery of adjuvant radiotherapy both substantially influence patient outcomes (16).(ii)Strengthening pathology and diagnostic infrastructure, including immunohistochemistry and molecular testing for rarer histologic subtypes. This strategy improves diagnostic accuracy, facilitates appropriate treatment allocation, and enriches histologic data submitted to cancer registries (30). (iii) Establishing or consolidating multidisciplinary head and neck tumour boards together with supporting referral pathways. This reduces care fragmentation, which disproportionately affects patients with late presentation, and can be implemented at relatively low cost in settings without available capital investment (31). Expansion of cancer registration and routine reporting of MIR and PMR serve as a cross-cutting prerequisite across all three strategies (32).

Measurement is a constraint that deserves emphasis in its own right. Since MIR and PMR are the only survival proxies available for this analysis, and both are vulnerable to under-ascertainment, the observed HDI-associated differences may reflect variation in measurement quality just as much as disparities in care quality. Population-based cancer registration, which provides the incidence denominator, covers approximately one-quarter to one-third of the global population, with coverage varying by registry and year (23, 24). Coverage is lowest in low-HDI settings where under-ascertainment is most severe, and is particularly sparse across Africa and Asia. Without improved cancer registration, the survival deficits suggested by these ratios cannot be quantified, still less tracked over time. Investment in cancer registration is relatively low-cost per recorded case and directly supports the prevention, screening and treatment planning required by national cancer control plans (32). Prioritizing surveillance infrastructure will allow future iterations of this analysis to replace modelled ratios with observed survival, the patient-relevant outcome of greatest importance (33).

Two cautions follow from the projections, and they bear on different questions rather than pulling in opposite directions. The steepest relative increases occur in Mexico and Brazil, yet these are also the settings whose absolute burden stays small, so a growth percentage should not be taken as grounds for moving resources away from the largest absolute contributors, China and India (13). At the same time, these tumors peak at older ages, the demand they generate will land on services already carrying a rising chronic-disease load, and folding head-and-neck assessment into existing geriatric and primary-care pathways is likely to prove more workable than setting up parallel rare-cancer programmers. The geographic pattern set out here is a starting point and not a final word: estimates of rare-cancer burden are only as reliable as the registries behind them, and the priorities above should be revisited as surveillance improves (33). These outputs are descriptive modelling results, not clinical forecasts: they show how population growth and aging would shift case and death counts if rates were held fixed, and they should not be read as predictions of what will happen or as guidance for the care of any individual patient.

Five inherent features of the dataset constrain the scope of conclusions that can be drawn from this study. GLOBOCAN estimates are modelled from registries and vital statistics of uneven quality, and it is in low- and medium-HDI settings that under-enumeration and inconsistent coding are most marked (14). Statistical comparisons call for caution in the absence of 95% UIs (Section 2.1). Each reported correlation comes with a bootstrap confidence interval, and those intervals are wide (Section 2.3). The back-filling procedure rests on the assumption that male and female counts sum to the both-sexes value; once the four reconstructed economies were excluded, the country-level correlation was non-significant and its sign reversed (Section 2.5). The demographic-only projection holds 2024 rates fixed, and the APC scenarios are deterministic single-parameter extrapolations (Section 2.4). The design, finally, is ecological: associations fitted to aggregate rates are open to ecological bias and cannot be carried over to individuals (1, 21, 34), and the same caution applies whenever a gradient seen across the four global HDI aggregates is read as a property of member countries (22). The four aggregate rows and nineteen country-level rows represent distinct populations at different scales, and the manuscript maintains strict separation between them throughout. Country-level estimates cannot be inferred from aggregate data, and vice versa. Pooling cases at the top anatomical site level masks underlying histologic heterogeneity.

## Data Availability

The original contributions presented in the study are included in the article/**Supplementary Material**. Further inquiries can be directed to the corresponding author.
