# Supplementary material for: Global burden, geographic and sex disparities, and projections to 2050 for salivary gland cancer across G20 countries: a GLOBOCAN 2024 analysis
Source: Front Oncol. 2026 Oct 26;16:1995714. doi: 10.3389/fonc.2026.1995714 (PMC13745338; doi:10.3389/fonc.2026.1995714)
Supplement: Supplementary file 1 [file Table1.docx]

**Supplementary Material**

**Table S1** Human Development Index (2022) for the 19 G20 member economies, used for the G20-restricted HDI dichotomization.

| **Group** | **Country** | **HDI (2022)** | **Position vs G20 median (0.875)** |
| --- | --- | --- | --- |
| Higher-HDI (n = 10) | Germany | 0.950 | ≥ median |
| Higher-HDI (n = 10) | Australia | 0.946 | ≥ median |
| Higher-HDI (n = 10) | United Kingdom | 0.940 | ≥ median |
| Higher-HDI (n = 10) | Canada | 0.935 | ≥ median |
| Higher-HDI (n = 10) | Republic of Korea | 0.929 | ≥ median |
| Higher-HDI (n = 10) | United States | 0.927 | ≥ median |
| Higher-HDI (n = 10) | Japan | 0.920 | ≥ median |
| Higher-HDI (n = 10) | France (metropolitan) | 0.910 | ≥ median |
| Higher-HDI (n = 10) | Italy | 0.906 | ≥ median |
| Higher-HDI (n = 10) | Saudi Arabia | 0.875 | ≥ median |
| Lower-HDI (n = 9) | Türkiye | 0.855 | < median |
| Lower-HDI (n = 9) | Argentina | 0.842 | < median |
| Lower-HDI (n = 9) | Russian Federation | 0.821 | < median |
| Lower-HDI (n = 9) | China | 0.788 | < median |
| Lower-HDI (n = 9) | Mexico | 0.781 | < median |
| Lower-HDI (n = 9) | Brazil | 0.760 | < median |
| Lower-HDI (n = 9) | South Africa | 0.717 | < median |
| Lower-HDI (n = 9) | Indonesia | 0.713 | < median |
| Lower-HDI (n = 9) | India | 0.644 | < median |

Note：Higher-HDI group (n = 10, HDI ≥ G20 median = 0.875）: Germany 0.950, Australia 0.946, United Kingdom 0.940, Canada 0.935, Republic of Korea 0.929, United States 0.927, Japan 0.920, France 0.910, Italy 0.906, Saudi Arabia 0.875. Lower-HDI group (n = 9, HDI < G20 median): Türkiye 0.855, Argentina 0.842, Russian Federation 0.821, China 0.788, Mexico 0.781, Brazil 0.760, South Africa 0.717, Indonesia 0.713, India 0.644. The higher- and lower-HDI groups defined by this median split are the ones used for the G20-restricted comparisons in the main text; the classification is listed here so that it can be reproduced exactly. Values are the 2022 UNDP Human Development Index as recorded in the GLOBOCAN 2024 country-level export used for this analysis.

**Table S2** Full indicator set for the 19 G20 member economies in 2024 (both sexes, all ages), listed in descending order of age-standardized incidence rate (ASIR).

| **Country** | **Incident cases** | **ASIR** | **Deaths** | **ASMR** | **5-yr prevalent cases** | **Prev. rate** | **MIR** | **PIR** | **PMR** |
| --- | --- | --- | --- | --- | --- | --- | --- | --- | --- |
| Canada | 695 | 0.86 | 165 | 0.15 | 2,456 | 6.18 | 0.24 | 3.53 | 14.88 |
| Italy | 1,126 | 0.84 | 433 | 0.20 | 3,879 | 6.54 | 0.38 | 3.44 | 8.96 |
| United States of America | 5,420 | 0.84 | 1,171 | 0.14 | 19,243 | 5.57 | 0.22 | 3.55 | 16.43 |
| Australia | 387 | 0.80 | 127 | 0.17 | 1,411 | 5.28 | 0.33 | 3.65 | 11.11 |
| Indonesia | 2,355 | 0.75 | 1,278 | 0.40 | 6,706 | 2.37 | 0.54 | 2.85 | 5.25 |
| Korea, Republic of | 653 | 0.73 | 172 | 0.14 | 2,423 | 4.69 | 0.26 | 3.71 | 14.09 |
| Japan | 1,913 | 0.69 | 738 | 0.17 | 6,719 | 5.43 | 0.39 | 3.51 | 9.10 |
| Argentina | 429 | 0.67 | 127 | 0.17 | 1,409 | 3.08 | 0.30 | 3.28 | 11.09 |
| France (metropolitan) | 890 | 0.65 | 305 | 0.14 | 3,056 | 4.59 | 0.34 | 3.43 | 10.02 |
| United Kingdom | 737 | 0.58 | 311 | 0.16 | 2,685 | 3.88 | 0.42 | 3.64 | 8.63 |
| Russian Federation | 1,447 | 0.57 | 655 | 0.25 | 4,724 | 3.26 | 0.45 | 3.26 | 7.21 |
| Brazil | 1,577 | 0.54 | 508 | 0.16 | 4,681 | 2.21 | 0.32 | 2.97 | 9.21 |
| Germany | 1,107 | 0.53 | 444 | 0.16 | 3,910 | 4.62 | 0.40 | 3.53 | 8.81 |
| Türkiye | 575 | 0.51 | 153 | 0.13 | 1,977 | 2.26 | 0.27 | 3.44 | 12.92 |
| China | 10,676 | 0.47 | 3,735 | 0.14 | 33,716 | 2.40 | 0.35 | 3.16 | 9.03 |
| Mexico | 671 | 0.46 | 193 | 0.12 | 2,084 | 1.59 | 0.29 | 3.11 | 10.80 |
| India | 6,791 | 0.45 | 2,033 | 0.14 | 17,842 | 1.23 | 0.30 | 2.63 | 8.78 |
| South Africa | 276 | 0.43 | 78 | 0.13 | 799 | 1.25 | 0.28 | 2.89 | 10.24 |
| Saudi Arabia | 87 | 0.30 | 35 | 0.13 | 315 | 0.93 | 0.40 | 3.62 | 9.00 |
| G20 total (19 economies) | 37,812 |  | 12,661 |  | 120,035 |  | 0.33 | 3.17 | 9.48 |

Note：ASIR, age-standardized incidence rate; ASMR, age-standardized mortality rate; MIR, mortality-to-incidence ratio; PIR, prevalence-to-incidence ratio; PMR, prevalence-to-mortality ratio. ASIR and ASMR are per 100,000 person-years (World Standard Population); 5-year prevalence rate is per 100,000. All rates and ratios are shown to the precision published by the source (two decimals for ASIR/ASMR and for the derived ratios; one decimal for the 5-year prevalence rate). Source: GLOBOCAN 2024 (Cancer Today), both sexes, salivary glands (ICD-10 C07–C08).

**Table S3.** Mortality-to-incidence ratio (MIR) by country and sex, G20 member economies, GLOBOCAN 2024

| **Country** | **Male cases** | **Male deaths** | **Female cases** | **Female deaths** | **MIR male / female (difference)** |
| --- | --- | --- | --- | --- | --- |
| Indonesia | 1531 | 854 | 824 | 424 | 0.5578 / 0.5146 (+0.0432) |
| Russian Federation | 728 | 405 | 719 | 250 | 0.5563 / 0.3477 (+0.2086) |
| United Kingdom | 413 | 221 | 324 | 90 | 0.5351 / 0.2778 (+0.2573) |
| Saudi Arabia | 63 | 30 | 24 | 5 | 0.4762 / 0.2083 (+0.2679) |
| Italy | 662 | 284 | 464 | 149 | 0.4290 / 0.3211 (+0.1079) |
| Germany | 677 | 284 | 430 | 160 | 0.4195 / 0.3721 (+0.0474) |
| Japan | 1156 | 472 | 757 | 266 | 0.4083 / 0.3514 (+0.0569) |
| China | 6239 | 2453 | 4437 | 1282 | 0.3932 / 0.2889 (+0.1042) |
| France (metropolitan) | 535 | 201 | 355 | 104 | 0.3757 / 0.2930 (+0.0827) |
| India | 3965 | 1359 | 2826 | 674 | 0.3427 / 0.2385 (+0.1042) |
| South Africa | 148 | 49 | 128 | 29 | 0.3311 / 0.2266 (+0.1045) |
| Mexico | 359 | 117 | 312 | 76 | 0.3259 / 0.2436 (+0.0823) |
| Argentina | 254 | 80 | 175 | 47 | 0.3150 / 0.2686 (+0.0464) |
| Korea, Republic of | 363 | 112 | 290 | 60 | 0.3085 / 0.2069 (+0.1016) |
| Canada | 427 | 113 | 268 | 52 | 0.2646 / 0.1940 (+0.0706) |
| Türkiye | 317 | 81 | 258 | 72 | 0.2555 / 0.2791 (-0.0235) |
| United States of America | 3232 | 760 | 2188 | 411 | 0.2351 / 0.1878 (+0.0473) |

Note：Not computable from the 2024 GCO export: Australia, Brazil. MIR = all-age deaths / all-age incident cases, computed separately for males and females directly from the 2024 GCO export without intermediate rounding. Countries are ordered by male MIR. The final column gives the male minus female difference. Australia and Brazil are not shown because the export does not publish both the male/female incidence and the male/female mortality cells required to compute a sex-specific MIR for them; for Australia the female incidence cell is absent and for Brazil the male mortality cell is absent. This table supports the statement in Section 3.3 that male MIR exceeded female MIR in 16 of the 17 economies for which the ratio was computable, the sole exception being Türkiye. Source: GLOBOCAN 2024.Male MIR was higher than female MIR in 16 of the 17 computable economies. The sole exception was Türkiye (male 0.2555 vs female 0.2791). The values are unrounded all-age death/case ratios computed directly from the export; differences below 0.05 occur in 5 of the 17 economies and are reported as observed rather than used to claim a stable sex ordering.
